# Visual Outcomes after Implantation of Lucidis EDOF IOL

**DOI:** 10.1155/2022/5100861

**Published:** 2022-05-28

**Authors:** Mark Rabinovich, Gaia Ceresara, Ana Aramburu del Boz, Danial Al Khatib, Marie Crespe, Jerome Bovet

**Affiliations:** Ophthalmology Network Organisation, Clinique de L'Oeil SA, Onex, Geneva, Switzerland

## Abstract

**Purpose:**

To evaluate the visual performance and clinical outcomes after implantation of Lucidis EDOF IOL following cataract surgery.

**Design:**

In this retrospective study, medical records from all enrolled patients were analyzed, and the following information was extracted retrospectively over 3 months following surgery.

**Materials and Methods:**

We reviewed retrospectively 181 eyes of 98 patients, who underwent cataract surgery with Lucidis extended depth of focus IOL.

**Results:**

44 patients were males (45%) and 54 were females (55%). The average age of the study population was 68 ± 11 years. The mean preoperative BCVA (logMAR) was 0.19 ± 0.18. The mean root mean square (RMS) high order aberration (HOA) was 0.18 ± 0.1. Monocular BCVA results were 0.02 ± 0.04 (logMAR) and 0.028 ± 0.04 (logMAR) 1 month and 3 months postoperatively, respectively. Between the baseline and 1-month measures, monocular distance BCVA improved by an average of 0.17 ± 0.14 logMAR (*p* = 0.0001). Between the baseline and 3-month postoperative measures, monocular distance BCVA improved by an average of 0.16 ± 0.13 logMAR (*p* = 0.0001). Monocular UDVA 1 and 3 months postoperatively was 0.08 ± 0.1 logMAR and 0.067 ± 0.08 logMAR, respectively. 1-Month postoperative binocular UDVA was 0.036 ± 0.05 logMAR, binocular UIVA was 0.1 ± 0.08 logMAR, and binocular UNVA was 0.12 ± 0.14 logMAR. 3-Month postoperative binocular UDVA was 0.038 ± 0.05 logMAR, binocular UIVA was 0.09 ± 0.1 logMAR, and binocular UNVA was 0.16 ± 0.14 logMAR.

**Conclusions:**

Lucidis EDOF IOL achieves good visual performances in all distances.

## 1. Introduction

Cataract surgeons are constantly in search of better refractive postoperative outcomes and better visual performances. New designs of intraocular lens (IOLs) help to achieve a more optimal visual performance and patient satisfaction.

Normally, the surgeons treat cataract patients by replacing the lens with a monofocal posterior chamber IOL. Therefore, patients should make a decision preoperatively which range of vision they prioritize postoperatively.

The introduction of the newest IOLs with extended depth of focus (EDOF) technology enabled a relatively good visual performance in intermediate distances as well as for far and near distances [1–6]. These ranges of distance are usually important for patients who perform activities such as using an iPad or a computer.

Furthermore, EDOF IOLs have been found to be associated with fewer halos and less contrast sensitivity loss than traditional multifocal IOL technology [7].

The basic principle of such IOLs is to create a single-elongated focal point to enhance the depth of focus, to eventually eliminate the overlapping of near and far images, as seen with other IOLs [8].

The Swiss innovation of the Lucidis IOL (Swiss Advanced Vision, SAV‐IOL SA, Neuchâtel, Switzerland) is a new type of refractive/EDOF hybrid IOL involving a central aspheric element surrounded by an outer refractive ring [9].

The central aspheric zone in an area of 1 mm acts as an axicon, so that the emerging light forms a Bessel beam, resulting in a beam of focal fields that allow a continuous vision from intermediate to short distances. This aspheric geometry does not generate any additional spherical aberration to the lens.

The aim of the current study is to evaluate the visual performance and clinical outcomes with Lucidis EDOF IOL.

## 2. Patients and Methods

This is a single-center, retrospective study of 98 patients (181 eyes) who underwent implantation of the Lucidis EDOF IOL (Swiss Advanced Vision, SAV‐IOL SA, Neuchâtel, Switzerland) during cataract surgery by 4 experienced surgeons between 2019 and 2022.

We performed a monocular unilateral analysis of preoperative and postoperative best corrected visual acuity (BCVA) and monocular uncorrected distant visual acuity (UDVA) of each eye. Uncorrected near visual acuity (UNVA) and uncorrected intermediate visual acuity (UIVA) were analyzed bilaterally. UDVA was also analyzed bilaterally for the purpose of visual acuity reporting.

Institutional board approval of the study and patients' consents were obtained. No conflicts of interest to be reported for this study.

Exclusion criteria were the presence of a concomitant ophthalmic condition, including age-related macular degeneration, diabetic retinopathy, glaucoma, uveitis, corneal opacities, or astigmatism of >1.00 *D*, age <18 years, and no previous corneal surgery.

The decision to perform cataract surgery and the choice of the IOL were based on clinical indications.

### 2.1. Intraocular Lens

Lucidis is a single-piece foldable multizone refractive/aspheric IOL, with a 360° square edge design and closed-loop haptics. The lens has a 6.0 mm optical diameter and a total diameter of either 10.8 mm or 12.4 mm. It is made from hydrophilic acrylic with a 26% water content. Optically, the Lucidis IOL uses both refraction and an aspheric element—the Axicon. The 1 mm aspheric zone occupies the center of the IOL and is surrounded by a 6 mm refractive ring.

### 2.2. Surgery

All surgeries were performed under local anesthesia and a venous sedation with Rapifen and propofol on demand. All interventions were performed using the same standard protocol on a temporal side, 2 mm main incision, and phacoemulsification machine (Stellaris, Bausch USA); for all FLACS, a femto laser LDV Z8 (Ziemer Ophthalmic Systems, Switzerland) was used.

A foldable Lucidis IOL was implanted in the capsular bag. No Lucidis IOLs were implanted in the sulcus.

### 2.3. Follow-Up Visits

Patients attended follow-up visits as a local protocol, at 1 day, 7 days, 1 month, and 3 months postoperatively. Additional appointments were planned at the surgeon's discretion. At each of the follow-up visits, the results were recorded in the patients' medical notes. 1-Month postoperative follow-up was done for 181 eyes, and 3-month postoperative follow-up was available for 78 eyes.

For refractive purposes, we tested monocular best-corrected visual acuity (BCVA), monocular and binocular uncorrected distant visual acuity (UDVA) at 6 meters, and binocular uncorrected visual acuity for near and intermediate distances (UNVA and UIVA, respectively) at 40 cm and 80 cm in photopic conditions. The surgeons performed the visual acuity testing in the same room and with the same ambience illumination using a Snellen decimal chart. The ophthalmologists recorded their observations following a slit-lamp examination as well as any subjective complaint or adverse event.

### 2.4. Statistical Analysis

Statistical analysis was performed on all available 1-month data. Quantitative and qualitative variables are described in terms of sample size, mean, standard deviation, median, range, and missing data.

Excel (Microsoft, WA, USA) was used for data analysis. The following analyses were performed for all parameters: an independent *t*-test to compare two means, and a Wilcoxon test to compare nonparametric continuous data. Results were considered statistically significant if *p* < 0.05.

SPSS version 26 (IBM, USA) was used for the ANOVA test and regression analysis for independent risk factor identification. Results were considered statistically significant if *p* < 0.05.

## 3. Results

181 eyes of 98 patients were included, all of which had up to 3-month data. In the cohort, 44 patients were males (45%) and 54 were females (55%). The average age of the study population was 68 ± 11 years. The mean preoperative BCVA (logMAR) was 0.19 ± 0.18, as shown in Table 1. The mean root mean square (RMS) high order aberration (HOA) was 0.18 ± 0.1.

### 3.1. Postoperative Best-Corrected Visual Acuity

Monocular BCVA results were 0.02 ± 0.04 (logMAR) and 0.028 ± 0.04 (logMAR) 1 month and 3 months postoperatively, respectively. Between the baseline and 1-month postoperative measures, monocular distance BCVA improved by an average of 0.17 ± 0.14 logMAR (*p* = 0.0001). Between the baseline and 3-month postoperative measures, monocular distance BCVA improved by an average of 0.16 ± 0.13 logMAR (*p* = 0.0001).

### 3.2. Monocular Postoperative Uncorrected Visual Acuity

Monocular UDVA 1 and 3 months postoperatively was 0.08 ± 0.1 logMAR and 0.067 ± 0.08 logMAR, respectively.

### 3.3. Binocular Postoperative Uncorrected Visual Acuity

Binocular UCVA was recorded until 3 months postoperatively. 1-Month postoperatively, binocular UDVA was 0.036 ± 0.05 logMAR, binocular UIVA was 0.1 ± 0.08 logMAR, and binocular UNVA was 0.12 ± 0.14 logMAR. 3-Months postoperatively, binocular UDVA was 0.038 ± 0.05 logMAR, binocular UIVA was 0.09 ± 0.1 logMAR, and binocular UNVA was 0.16 ± 0.14 logMAR.

### 3.4. Postoperative Spherical Equivalent and Cylinder

1-Month postoperatively, 24% of the eyes had emmetropic spherical equivalents (SE) between 0.13 and +0.13*D*. The mean monocular UDVA for that range of SE was 0.07 ± 0.09 logMAR.

**Figure 1:**
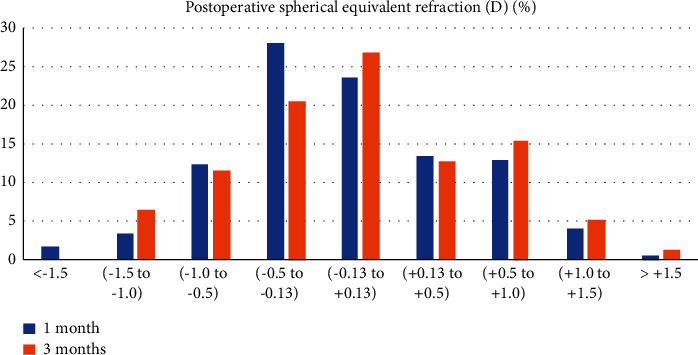
Postoperative spherical equivalent.

3 months postoperatively, 27% of the eyes had emmetropic spherical equivalents (SE) between 0.13 and + 0.13*D*, as shown in Figure 1. The mean monocular UDVA for that range of SE was 0.07 ± 0.09 logMAR.

1 month postoperatively, 57% of the eyes had a cylinder value <-0.5*D*. The mean monocular UDVA for that range of postoperative cylinder was 0.078 ± 0.09 logMAR.

**Figure 2:**
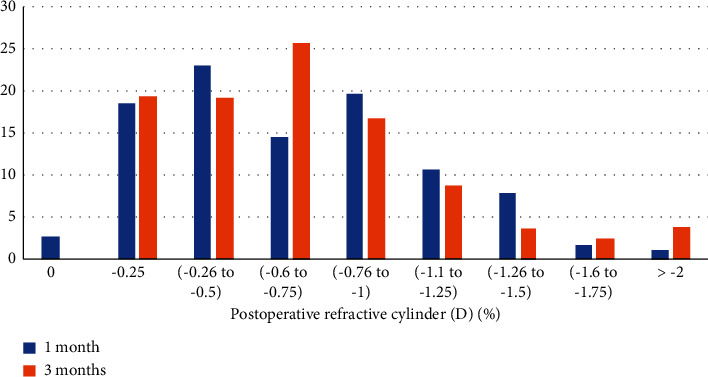
Postoperative cylinder.

3 months postoperatively, 38% of the eyes had a cylinder value <−0.5*D*, as seen in Figure 2. The mean monocular UDVA for that range of postoperative cylinder was 0.08 ± 0.1 logMAR.

Perioperative and postoperative negative outcomes included complications, glares, or halos reported by patients and discomfort with near vision that requiring spectacles or an eventual add-on operation, as seen in Table 2.

## 4. Discussion

In our study, we demonstrated good results for far, intermediate, and short uncorrected visual acuity for eyes after implantation of Lucidis EDOF IOLs up to 3 months postoperatively.

Nowadays, we are familiar with many types of IOLs with EDOF characteristics that were described under the term EDOF.

Among these are pure EDOF IOLs (e.g., TECNIS Eyhance) and EDOF IOLs using the pinhole effect (e.g., XtraFocus). There are also hybrid MF/EDOF IOLs that are further subdivided to diffractive/EDOF IOLs (e.g., TECNIS Symfony), refractive/EDOF IOLs (e.g., Lucidis IOLs), and refractive-diffractive/EDOF IOLs (e.g., EDEN, Harmonis, TECNIS Synergy) [8].

The particularity of the Lucidis IOL is the central aspheric zone in an area of 1 mm that acts as an axicon, resulting in a beam of focal fields that allow a continuous vision from intermediate to short distances. This aspheric geometry does not generate any additional spherical aberration to the lens.

Furthermore, the main benefit of the design of the Lucidis IOL is to provide additional visual comfort at near and intermediate distances [9, 10]. Notwithstanding the benefits, this IOL was found inferior for distant uncorrected visual acuity, compared to results of other EDOF IOLs [10].

EDOF IOLs (Mini Well; SIFI, Catania, Italy) were previously demonstrated to perform similarly to multifocal IOLs (ReSTOR SV25T; Alcon Laboratories, Inc., Fort Worth, TX) for far and near distances, with superiority at the intermediate level [11].

Compared to Trifocal IOLs (AT LISA tri 839MP IOL), for the intermediate distance visual performance, EDOF IOL (Symfony IOL) was also found superior [12].

EDOF IOLs were found to provide not only a subjective satisfaction but also a good quality of uncorrected postoperative visual acuities at all distances [13, 14].

In our study, monocular UDVA 1 and 3 months postoperatively was 0.08 ± 0.1 logMAR and 0.067 ± 0.08 logMAR, respectively.

In a meta-analysis of 24 studies, Rosen et al. demonstrated a mean UDVA of 0.11 logMAR, achieved after multifocal IOL implantation [15]. Hogarty et al. found a mean binocular UDVA for TECNIS Symfony ERV IOL (TECNIS Symfony; Abbott Medical Optics, Inc., Abbott Park, IL) of 0.04 logMAR [16]. In our study, for Lucidis EDOF IOLs, we found a comparable 1-month postoperative binocular UDVA of 0.036 ± 0.05 logMAR and 3-month postoperative binocular UDVA of 0.038 ± 0.05 logMAR.

Furthermore, in our study, we found binocular UIVA of 0.1 ± 0.08 logMAR and 0.09 ± 0.1 logMAR 1 and 3 months postoperatively, respectively. Our results for the intermediate uncorrected visual acuity were comparable to those reported by Cochener et al. for TECNIS EDOF IOL [17]. The authors showed the latter achieved a mean UIVA of 0.18 logMAR for the intermediate range.

In addition, we found that binocular UNVA was 0.12 ± 0.14 logMAR and 0.16 ± 0.14 logMAR, 1 and 3 months postoperatively, respectively, which were also comparable to previous reports of TECNIS Symfony IOL of 0.28 logMAR at the near range [17], the latter with a mean binocular VA (decimal) of 0.64 ± 0.07.

In our study, we also demonstrated an emmetropic spherical equivalent in 24% of the eyes (−0.13 and + 0.13 D) and 27%, 1 and 3-months postoperatively, respectively. The SE corresponded to a mean monocular UDVA of 0.07 ± 0.09 logMAR and 0.07 ± 0.09 logMAR, 1 and 3 months postoperatively, respectively. On the other hand, Cochener et al. found emmetropic SE outcomes for TECNIS Symfony (Abbott Medical Optics, Inc., Abbott Park, IL) for 8% of the eyes (17).

In addition, in our study, we demonstrated that 57% and 38% of the eyes had a cylinder value of less than −0.5 *D*, 1 and 3 months postoperatively, respectively. The corresponding mean monocular UDVA was 0.078 ± 0.09 logMAR and 0.08 ± 0.1 logMAR, 1 and 3 months postoperatively, respectively, while Cochener showed that, for TECNIS Symfony IOL, 56% of the eyes had a postoperative cylinder of less than 0.5 *D* (17).

In our study, only 5% of the patients complained of visual aberrations during the follow-up period, 4% complained of diplopia, 20% were provided with reading glasses for a suboptimal postoperative UNVA, and 3% opted for an additional Add-On IOL (HumanOptics AG, Erlangen, Germany), a line of sulcus-fixated silicone lenses.

However, it was previously shown that dysphotopsias such as halos were inevitable in EDOF IOLs and increase with higher near addition powers [18, 19].

Preoperatively, it is important to consider the specific patient's visual needs as well as the extent to which they are willing to accept such optical side effects.

If, for example, the patients would not tolerate dysphotopsias and were willing to accept glasses for reading, one possibility with EDOF IOLs could be micro monovision of −0.75 *D* anisometropia.

While our study focused on virgin eyes undergoing cataract surgery with Lucidis EDOF IOL implantation, future studies are warranted to evaluate dysphotopsia and spherical aberration incidence in eyes with a previous refractive surgery (e.g., radial keratotomy) that are scheduled to have cataract surgery and an EDOF IOL implantation. Any previous refractive surgery can be associated with a more challenging IOL power calculation, as well as a postoperative refractive surprise and corneal instability (postoperative astigmatism).

For such less convenient conditions for an EDOF IOL consideration, Meduri et al. suggested some perioperative methods such as stabilizing sutures that lowered the risk of dehiscence as well as the postoperative astigmatism [20, 21].

Furthermore, Baartman et al. found that for eyes with previous radial keratotomy that had undergone phacoemulsification with implantation of the TECNIS Symfony EDOF IOL, there were good visual outcomes and a subjective satisfaction of the patients [22].

The current study has several limitations. Firstly, its retrospective nature. Secondly, this study lacks long-term follow-up with only 3-month data available that does not allow for analysis of potential long-term complications.

The retrospective nature of the study did not allow to include corrected near and intermediate visual acuity which would have allowed the confirmation as to whether the previously documented performance of this IOL for distance vision was due to its design or to a myopic postoperative spherical equivalent. Nor did it allow to include a baseline UCVA measured preoperatively or the postoperative contrast perception.

In conclusion, in our study, we demonstrated good visual outcomes for Lucidis EDOF IOL for all ranges of vision. Furthermore, investigations of the long-term performance of this IOL is warranted in future studies.

## Figures and Tables

**Table 1:** Demographics of cohort.

Patients	*n* = 98 (181 eyes)
Male/female	44/54
Age	68 ± 11
Preoperative best-corrected visual acuity (logMAR)	0.19 ± 0.18
RMS HOA	0.18 ± 0.1

**Table 2:** Negative outcomes.

Negative outcomes	98 patients (%)
Capsular rupture	1
Diplopia	4
Eventual add-on	3
Eventual IOL change	1
Endophthalmitis	1
Halos/glare	5
Indication for reading glasses	20

## Data Availability

Data are available upon request.
